# L-Boronphenylalanine Biodistribution Dynamics in the Organs of Mice with Subcutaneous Tumor Xenograft is a Model to Assess Neuron Sources Efficiency in Boron Neutron Capture Therapy

**DOI:** 10.17691/stm2023.15.6.02

**Published:** 2023-12-27

**Authors:** I.N. Druzkova, A.A. Pakhomova, N.I. Ignatova, A.R. Suleymanova, A.V. Maslennikova

**Affiliations:** PhD, Researcher, Fluorescent Bioimaging Laboratory, Research Institute of Experimental Oncology and Biomedical Technologies; Privolzhsky Research Medical University, 10/1 Minin and Pozharsky Square, Nizhny Novgorod, 603005, Russia; Student; National Research Lobachevsky State University of Nizhny Novgorod, 23 Prospekt Gagarina, Nizhny Novgorod, 603022, Russia; PhD, Associate Professor, Department of Epidemiology, Microbiology and Evidence-Based Medicine; Privolzhsky Research Medical University, 10/1 Minin and Pozharsky Square, Nizhny Novgorod, 603005, Russia; Student; Privolzhsky Research Medical University, 10/1 Minin and Pozharsky Square, Nizhny Novgorod, 603005, Russia; MD, DSc, Head of the Department of Oncology, Radiation Therapy and Radiology; Privolzhsky Research Medical University, 10/1 Minin and Pozharsky Square, Nizhny Novgorod, 603005, Russia

**Keywords:** boron neutron capture therapy, L-boronphenylalanine, L-BPA, inductively coupled plasma mass spectroscopy, biodistribution, radiation therapy

## Abstract

Boron neutron capture therapy (BNCT), due to its high biological efficiency, is one of the most promising methods of radiation therapy for malignant tumors. Currently, research in this area has received momentum due to the emergence of fundamentally new compact neutron sources suitable for clinical use.

**The aim of the investigation** was to study L-boronphenylalanine (L-BPA) biodistribution in the organs of experimental animals with subcutaneous tumor xenografts, and evaluate the application of the experimental model to assess the effectiveness of new neutron sources.

**Materials and Methods:**

The experiments were carried out on BALB/c mice with subcutaneous xenograft of mouse adenocarcinoma CT26. L-boronphenylalanine in a molar excess of fructose was administered intravenously at a dose of 350 mg/kg, the organs under study were taken 1.5, 3, 6, and 24 h after drug administration. The content of the ^10^B isotope was analyzed using inductively coupled plasma mass spectroscopy (ICP-MS). The absence of toxic effects was verified pathomorphologically.

**Results:**

The maximum L-BPA content in the tumor was 142.0±4.41 μg/g 1.5 h after drug administration. The minimum therapeutic concentration of L-BPA in the tumor persists up to 5.4 h after drug administration. Among normal organs, the maximum content was observed in the kidneys, it is most likely being associated with the structural and functional features of the organ rather than the true content of L-BPA in the tissues. Histological studies revealed no structural disorders and dystrophic changes in tissues against the background of L-BPA introduction.

**Conclusion:**

The results of the study demonstrate the feasibility of the studied tumor model to evaluate the efficiency of new neutron sources for BNCT. The L-borophenylalanine content in the tumor and the time of maintaining the minimum therapeutic concentration appeared to be sufficient for effective BNCT. The high contrast of ^10^B accumulation relative to non-pathological tissues minimizes the possible side effects of BNCT.

## Introduction

Boron neutron capture therapy (BNCT) is a kind of binary radiotherapy, and one of the most effective and promising methods to treat malignancies as of today. The method is based on making use of the reactions occurring between ^10^B isotope nuclei and a flow of neutrons, their energy being in the range of 5**·**10^–3^–5**·**10^4^ eV.

One of the major problems hindering the large-scale clinical BNCT implementation is the lack of compact neutron radiation sources, which can be installed in a radiotherapy clinic. From the beginning of BNCT epoch, nuclear reactors were used to obtain thermal and epithermal neutron beams, and it drastically limited the implementation of the method in clinical practice. Beams were obtained on the basis of self-sustaining chain nuclear reaction of uranium-235 degradation. The spectrum of such neutrons has the energy up to 10 MeV, the average energy being about 2 MeV [1, 2]. However, currently, there have already appeared the prototypes of compact accelerating sources of various modifications; the prototypes enable to obtain neutron beams with energy close to that required for clinical use [3–9]. Along with that, the prototypes of D-D-neutron generators have been developed, which enable to obtain neutron beams with density, which appears to be record for compact systems [10, 11]. Thus, currently, BNCT has been given a new lease of life and is relevant again to solve the biological problems related to its implementation.

**Figure 1.**
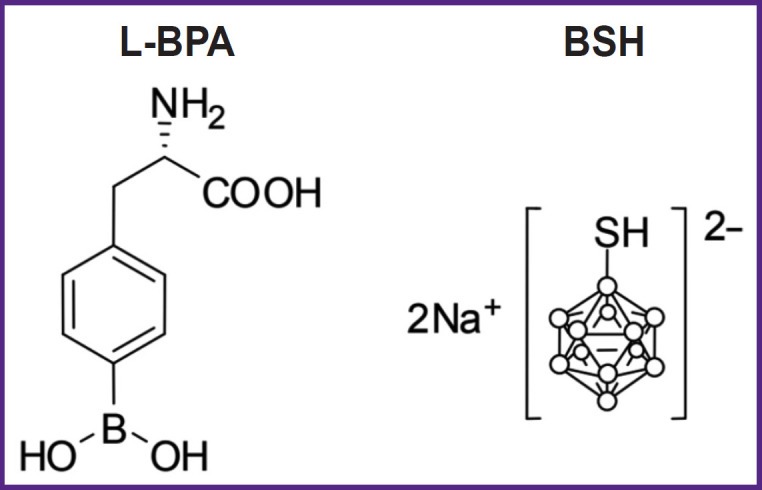
Structural formulas of L-BPA (L-p-dixydroxyboronanilphenylalanine, 4-(dihydroxyboranyl)-L-phenylalanine) and BSH (sodium mercaptoundecahydro-closododecaborate, sodium borocaptate) certified for clinical use as boron-containing agents

Boron preparations containing ^10^B atoms are the second component in BNCT. At present, there are only two preparations certified for clinical use: L-p-dixydroxy-boronanilphenylalanine (4-(dihydroxyboranyl)-L-phenylalanine, L-BPA) and sodium mercaptoundecahydro-closododecaborate (sodium borocaptate, BSH), their structural formulas are given in Figure 1.

Preparation L-BPA is an amino acid, which is capable of relatively selectively (maximum contrast 2:1) accumulating in a tumor due to the more effective transport system of L-amino acids in its cells compared to normal ones [12, 13]. The compound contains only one boron atom, although due to the low toxicity of the compound in general it enables to accumulate a sufficient for therapy amount of boron in the tumor cells. In BSH, there are 12 boron atoms, which enables with a great probability to achieve the necessary therapeutic concentration (24–35 μg/g) in tumor cells [14]; however, it has less accumulation contrast (1.1–1.8:1) compared to L-BPA [15].

It follows that the control over isotope ^10^B accumulation efficiency in a target-tumor, as well as in surrounding healthy tissues, is of great importance for BNCT development.

The highest accuracy of ^10^B isotope content estimate is achieved by using direct methods such as gamma ray spectroscopy [16–18], inductively coupled plasma mass spectrometry (ICP-MS) [19, 20], high-resolution alpha-radiography, alpha-ray spectroscopy, neutron capture radiography [21, 22], secondary neutral mass-spectrometry [23], spectroscopy of characteristic electronic energy loss [24], ion trap mass spectrometry [25]. Using the mentioned techniques, we received the data on ^10^B isotope accumulated in a tumor and in some tissues and organs. However, there are no detailed studies on ^10^B isotopes content dynamics in a tumor and vital organs, which can become a target for neutrons.

The selection of an adequate tumor model to assess BNCT effect is a critical task.

### The aim of the investigation

The present study offers a model of a tumor subcutaneous xenograft obtained from a cell line of CT26 intestinal adenocarcinoma in BALB/c mice as a potential model to assess the effectiveness of new neutron sources. The biodistribution of L-boronphenylalanine in organs and tissues of experimental animals was studied in dynamics by ICP-MS method and the prospects of using the presented model to evaluate the efficiency of new neutron sources application were determined.

## Materials and Methods

### Preparation of L-boronphenylalanine solution in molar fructose excess

L-BPA preparation for the experiments was given by the staff members of Budker Institute of Nuclear Physics of Siberian Branch Russian Academy of Sciences (Novosibirsk, Russia). The preparation solution in molar fructose excess was made according to the protocol by Coderre et al. [26]: we prepared weighed L-BPA portion to obtain the solution, its final concentration being 35 mg/ml, and fructose weighed portion at the rate of L-BPA:fructose=1:1.1. Weighed portion of L-BPA was dissolved in water purified using MilliQ system (Milliport, Germany) resulted in obtaining cloudy solution, its initial pH was 4.5–6.0. Then, using 10N of NaOH solution, pH was made to reach 9.0–10.5. The resulted solution was stirred for 20 min till it became clear by adding a fructose portion and stirring for at least 10 min to form a complex of L-BPA and fructose. Subsequently, using concentrated 1 M HCl, pH was gradually made to reach the value of 7.4 avoiding solution precipitation. The prepared solution was used on the day of preparation or stored at 4°C for no more than 12 days. Before use, the solution was filtered using an antibacterial filter, its pore diameter was 0.2 μm.

### The experiments on animals

The experiments were carried out on BALB/c line mice. A subcutaneous tumor was induced in the animals by CT26 cell suspension, the concentration was 5**·**10^5^ cells in 100 μl of phosphate buffer solution, which was injected in the right thigh area. When a tumor grew up to about 50 mm^3^, L-BPA preparation in a saturated fructose solution in a dose of 350 mg/kg body mass was administered intravenously to the animal’s lateral caudal vein. The study was approved by the Ethical Committee of Privolzhsky Research Medical University (Nizhny Novgorod, Russia), protocol No.19 dated December 9, 2022.

For tissue retrieval, the animals were sacrificed by a mixture of Zoletil (a dose of 40 mg/kg) and 2% Rometar (a dose of 10 mg/kg) followed by the euthanasia by cervical dislocation. The animal tissue samples of liver, kidneys, spleen, intestine, lungs, tumor, skin, muscular tissue, and blood were withdrawn 1.5, 3, 6, and 24 h after the L-BPA administration, at least 100 mg in volume. The samples were kept in labelled test tubes and stored at –80°C before the investigation. At every time point, tissue samples were withdrawn from three animals (n=12). By analogy, we analyzed L-BPA biodistribution in tumor-free animals (n=12). The study involved totally 24 animals.

### Determination of ^10^B isotope content using inductively coupled plasma mass spectroscopy

This study part was performed in the Institute of Microelectronics Technology and High-Purity Materials of Russian Academy of Sciences (Chernogolovka, Russia).

#### Decomposition of tissue samples

The procedure was performed in an autoclave decomposition system with resistance heating. Weighed portions of the samples under study, their mass being from 60 to 200 mg, were put into Teflon autoclave reaction tanks, added 2 ml of concentrated nitric acid — HNO_3_, 65% (Merk, Germany), and kept for 20 min at 160°C and for 1 h at 200°C.

#### Inductively coupled plasma mass spectroscopic analysis

The content of ^10^B and ^11^B isotopes, as well as ^9^Be and ^115^In (used as internal standards to account temporal variations of mass-spectrometer sensitivity and consider a matrix effect) in the samples was determined using XSeries 2 mass-spectrometer (Thermo Fisher Scientific, USA), it has the following parameters: generator output capability of 1350 W; concentric PolyCon (Glass Expansion) as a dispenser; spraying chamber — quartz cooled (3°C); plasma-forming argon flow consumption of 13 L/min; auxiliary argon flow consumption — 0.89 L/min; studied sample consumption — 0.8 ml/min; resolution of 0.8 M.

The isotopes were determined in the samples by using external calibration by calibrating solutions containing from 1 to 500 μg/L of analytes; among them, there were multiple-element standard solution ICP-MS-68; solution A; multiple-element standard solution ICP-AM-6A; certified reference material “Traces of metals in drinking water”, Standard B CRM-TMDW-B (all solutions manufactured by High-Purity Standards, USA).

### Statistical data processing

The data were presented as an arithmetic mean by three dimensions ± mean root square deviation. The tumor–blood and tumor–organs concentration ratio (accumulation contrast) was calculated as the ratio of tumor ^10^B content to its content in the studied organs and tissues (blood). When calculating the time of achieving minimal therapeutic concentration for a curve of ^10^B content decrease in a tumor, there was developed a trend line with max *R*^2^ (power dependence), *t* (time) was calculating by the formula:

t=[Cmin/252.41][1/(−1.394)],

where *C*_min_ — minimal therapeutic concentration, equal to 24 μg/g.

## Results

In the course of our work in order to reveal L-BPA biodistribution, we studied isotope ^10^B content using ICP-MS in the following animal tissues and organs: liver, kidneys, spleen, intestine, lungs, skin, muscular tissue, blood, and tumor (if any). Organ and blood samples were taken 1.5, 3, 6, and 24 h after intravenous L-BPA infusion.

### Biodistribution of L-boronphenylalanine in the organs of the animals with induced tumor

Table 1 presents the mean ^10^B isotope in the studied tissues in the animals with induced tumor.

**T a b l e 1:** ^10^B isotope content in the organs and tissues of the animals with induced tumor after L-boronphenylalanine administration

Organ/tissue	^10^B content (μg/g)			
	1.5 h	3 h	6 h	24 h
Tumor	142.0±4.41	59.30±3.63	18.67±4.0	3.10±0.01
Lung	45.0±1.40	18.90±1.16	6.57±1.41	<DL
Liver	31.50±0.98	15.13±0.93	5.33±1.14	<DL
Kidney	107.30±3.34	35.70±2.19	11.60±2.49	0.82±0.01
Spleen	50.77±1.58	25.13±1.54	6.90±1.48	<DL
Intestine	39.73±1.24	30.57±1.87	7.87±1.69	0.59±0.01
Muscle	67.40±2.10	25.37±1.55	7.50±1.61	0.75±0.01
Skin	73.17±2.27	28.70±1.76	9.70±2.08	<DL
Blood	32.17±1.0	16.33±1.0	4.67±1.0	<DL

N o t e: DL — determination limit.

**Figure 2.**
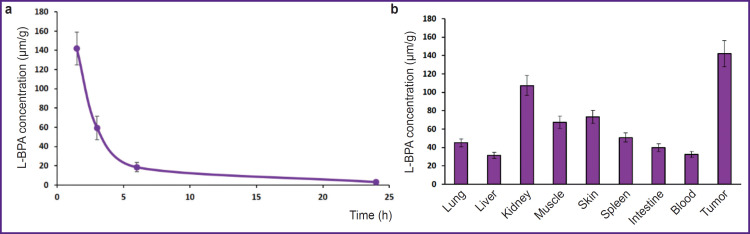
^10^B isotope content in the organs and tissues of the animals with induced tumor after L-boronphenylalanine administration: (a) ^10^B content decrease dynamics in a tumor; (b) ^10^B distribution in animal organs 1.5 h after L-boronphenylalanine administration

Figure 2 demonstrates the curve showing ^10^B isotope content decrease in a tumor depending on a time-point after drug infusion, also the diagram demonstrating ^10^B isotope concentration in the organs and tissues 1.5 h after the drug administration.

1.5 h after L-BPA administration, tumor–blood concentration ratio of ^10^B isotope was 4.4, while concentration ratios of tumor and normal tissues were from 1.3 to 4.5 (Table 2). Whereas ^10^B isotope content in a tumor significantly exceeded the required (according to literature data) therapeutic concentration (24–35 μg/g).

**T a b l e 2:** ^10^B isotope contrast accumulation in a tumor

Organ/tissue	In relation to an organ		In relation to blood	
	1.5 h	3 h	1.5 h	3 h
Tumor	1	1	4.41	3.63
Lung	3.15	3.14	1.4	1.16
Liver	4.51	3.92	0.98	0.93
Kidney	1.32	1.66	3.34	2.19
Spleen	2.8	2.1	1.58	1.54
Intestine	3.57	1.94	1.24	1.87
Muscle	2.11	2.34	2.1	1.55
Skin	1.94	2.1	2.27	1.76
Blood	4.41	3.63	1	1

In approximation of the ^10^B isotope content decrease curve in a tumor and non-pathological organs, we calculated the time to reach minimal required therapeutic concentration (24 μg/g). Minimal concentration in a tumor was found to be achieved 5.4 h after the L-BPA administration, while for most inner organs except skin and kidneys this time appeared to be from 1.0 to 2.6 h. Therefore, the optimal time for tumor irradiation was estimated in the range from 1.5 to 5.5 h after the drug administration.

### Biodistribution of L-boronphenylalanine in the organs of the animals without tumor

The study of L-BPA biodistribution in the organs and tissues of the animals without tumor showed ^10^B isotope content 1.5 h after the drug administration was the same as in the animals with tumor; however, the decreasing of ^10^B isotope concentration was slightly faster. Table 3 demonstrates mean ^10^B isotope content in the organs of the tumor-free animals.

**T a b l e 3:** ^10^B isotope content in the organs and tissues of the animals without tumor after L-boronphenylalanine administration

Organ/tissue	10B content (μg/g)			
	1.5 h	3 h	6 h	24 h
Lung	45.20±7.10	13.53±3.04	3.73±1.45	<DL
Liver	27.80±3.97	12.05±2.09	3.40±1.22	<DL
Kidney	59.73±4.16	28.80±9.43	9.27±4.37	<DL
Spleen	46.83±1.38	19.77±4.72	5.13±2.18	<DL
Intestine	36.37±5.06	14.87±3.13	3.93±1.27	<DL
Muscle	39.20±6.75	17.87±9.21	3.10±1.76	<DL
Skin	76.93±17.24	22.0±8.69	4.53±2.23	<DL
Blood	27.37±3.15	11.13±1.86	3.03±1.26	<DL

N o t e: DL — determination limit.

### Histological study

**Figure 3.**
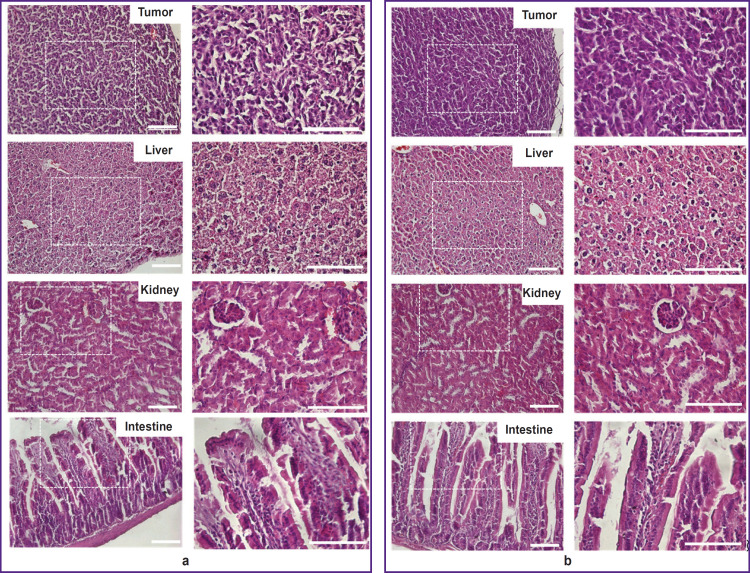
Representative histological images of the mice organs and tumor: (a) no L-BPA administration; (b) after L-BPA administration. Bar — 200 μm for all images, the area marked by dotted line was photographed with higher magnification (on the right panel)

We collected the samples of some organs and tissues to verify the absence of L-BPArelated toxic effect in mice organs. Pathomorphological study revealed neither structural disturbances nor cell degeneration signs in the tissues and organs of the animals with subcutaneous tumor xenograft after L-BPA administration compared to the control animals, who were not injected with L-BPA (Figure 3).

## Discussion

Using ICP-MS, maximal content of ^10^B isotope in a tumor (142.0±4.41 μg/g) was determined 1.5 h after L-BPA administration, tumor–blood concentration ratio of ^10^B isotope was 4.4, while concentration ratios of tumor and normal tissues were from 1.3 to 4.5. The required therapeutic concentration of ^10^B isotope in a tumor persisted up to 5.5 h after the drug administration, the accumulation contrast sustaining 3.63 in relation to blood and 1.66–3.92 in relation to other organs.

The present characteristics are one of the highest values represented in literature. In the study by Seo et al. [27] for a model of subcutaneous heterotopic tumor U87 (human glioma) in intravenous L-BPA administration at a dose of 500 μg/kg, ^10^B isotope content 1 h after injection was 23.7±5.1 μg/g, the tumor–blood concentration ratio being 2.8. For a model DLD-1 (colorectal cancer) in the retroperitoneal space in intra-abdominal injection of L-BPA at a dose of 250 mg/kg, the accumulation in a tumor reached its maximum 4 h after the injection and was 123.6±29.9 μg/g, the tumor–blood concentration ratio was 8.69±3.24 [28]. In the study by Yoshimura et al. [29], when forming lymphoma in the murine brain after intratumoral injection of L-BPA, 24 mg/kg, ^10^B content in a tumor was 9.9±1.6 μg/g, the tumor– blood concentration ratio being 0.7. Tsygankova et al. [30] studied the model of subcutaneous glioma U87: L-BPA was administered at a dose of 350 mg/kg in the retro-orbital sinus; ^10^B isotope content in the tumor 2 h after the injection was 11.0±2.0 μg/g, the tumor– blood concentration ratio being 1.6. Lee et al. [31] had the same experimental model; 1 h after intravenous injection, ^10^B isotope content was determined as being 152.2±12.1 μg/g. These data have proved higher efficiency of intravenous administration compared to that injected in the retro-orbital sinus. However, this study did not show ^10^B isotope biodistribution in other animal organs.

It should be noted that higher isotope ^10^B content compared to other healthy tissues was found in kidneys; however, it was likely to be related to functional and structural characteristics of kidneys as a filtering organ, since filter structures comprise the most of the parenchyma: tubules filtering the apparatus (Bowman’s capsule), as well as renal pelvises, where primary filtrate accumulates, which is in fact blood plasma. The values show ^10^B isotope content in biological fluids rather than in tissues. Relatively high ^10^B isotope content was also found in skin that is related, in particular, to active absorption of L-BPA by melanocytes, since its chemical structure is similar to tyrosine, which is necessary for melanogenesis [32].

Thus, in our research we studied for the first time L-BPA biodistribution in the organs of the animals with induced tumor, the dynamics was up to 24-h follow-up. The findings demonstrate a model tumor can be exposed to irradiation 1.5 h after the drug administration, when ^10^B isotope content in the tumor is about 140 μg/g; and irradiation is possible to last within at least 4 h. At the same time, throughout the radiation period in a tumor a minimal required concentration of ^10^B isotope will persist to perform BNCT, while accumulation contrast in relation to blood will be maintained at the level of 4.4–3.5, which will make it possible to achieve a therapeutic effect.

## Conclusion

The obtained results of L-boronphenylalanine biodistribution dynamics demonstrate the tumor model (subcutaneous xenograft of CT26 in BALB/c mice) feasibility to assess the efficiency of new neutron sources for boron neutron capture therapy. L-boronphenylalanine was found to sufficiently accumulate in a tumor needed for performing effective treatment and sustaining minimal therapeutic concentration within long time period, with good accumulation contrast in relation to non-pathological organs that enables to minimize possible side effects of boron neutron capture therapy.
